# Morphological and anatomical adaptations to dry, shady environments in *Adiantum reniforme* var. *sinense* (Pteridaceae)

**DOI:** 10.7717/peerj.9937

**Published:** 2020-09-30

**Authors:** Di Wu, Linbao Li, Xiaobo Ma, Guiyun Huang, Chaodong Yang

**Affiliations:** 1 Rare Plants Research Institute of Yangtze River, Three Gorges Corporation, Yichang, China; 2 Engineering Research Center of Ecology and Agriculture Use of Wetland, Ministry of Education, Yangtze University, Jingzhou, China

**Keywords:** Endodermis, Dictyostele, Sclerenchyma layer, Suberin lamellae, Thin cuticle

## Abstract

The natural distribution of the rare perennial fern *Adiantum reniforme* var. *sinense* (Pteridaceae), which is endemic to shady cliff environments, is limited to small areas of Wanzhou County, Chongqing, China. In this study, we used brightfield and epifluorescence microscopy to investigate the anatomical structures and histochemical features that may allow this species to thrive in shady, dry cliff environments. The *A. reniforme* var. *sinense* sporophyte had a primary structure and a dictyostele. The plants of this species had an endodermis, sclerenchyma layers and hypodermal sterome, reflecting an adaption to dry cliff environments. Blades had a thin cuticle and isolateral mesophyll, suggesting a tolerance of shady environments. These characteristics are similar to many sciophyte ferns such as *Lygodium japonicum* and *Pteris multifida*. Thus, the morphological and anatomical characteristics of *A. reniforme* var. *sinense* identified in this study are consistent with adaptations to shady, dry cliff environments.

## Introduction

*Adiantum reniforme* var. *sinense* (Pteridaceae, subfamily Vittarioideae) is a rare cliff-dwelling perennial pteridophyte, with a natural distribution limited to small areas of Wanzhou County, Chongqing, China. This plant has been used in Chinese medicine for more than 100 years (Lin, 1980; Zhang & Wu, 2013; Rothfels & Schuettpelz, 2014; PPG, 2016; Pryer et al., 2016). *A. reniforme* is of immense botanical interest due to its intercontinental distribution; *A. reniforme* is found in the Azores, *A. reniforme* var. *sinense* in China and *A. reniforme* var. *asarifolium* in south-central Africa (Lin, 1980; Zhang & Wu, 2013; Wang et al., 2015). The Chinese variants of this species have low genetic diversity (Pan, Ji & Chen, 2005; Liu, Gituru & Chen, 2007; Wang et al., 2015). In China, the natural habitat of this plant was lost due the construction of Three Gorges Dam, which was completed in 2012. Since this time, *A. reniforme* var. *sinense* has been conserved ex situ in the germplasm resource nursery managed by the China Three Gorges Corporation and the Wuhan Botanical Garden (Pan, Ji & Chen, 2005; Liao et al., 2007; Zhou, Jiang & Huang, 2008; Wu, 2012). Due to its endangered status, narrow distribution, ex situ conservation, and low genetic diversity, *A. reniforme* var. *sinense* is listed as a class II protected fern in China (Lin, 1980; Xu, Zhen & Jin, 1987; Fu & Jin, 1992; Pan, Ji & Chen, 2005; Liu, Gituru & Chen, 2007; Zhang et al., 2013; Wang et al., 2015).

Pteridophytes have evolved various morpho-anatomical features to adapt to terrestrial, xeric, epiphytic, rupicolous and aquatic environments (Wylie, 1949; Zhang et al., 2013; Neira et al., 2017; Sârbu et al., 2017; Wetzel et al., 2017); this group is thus taxonomically, evolutionarily, and phylogenically diverse (Kato & Imaichi, 1997; Li et al., 2013; Vasco, Moran & Ambrose, 2013; Plackett, Di Stilio & Langdale, 2015; Harrison & Morris, 2017; Huiet et al., 2018). Pteridophyte rhizomes have species-or group-specific stele, including the dictyostele of the Monachosorideae and the Polypodiaceae (Jeffreys, 1903; Wardlaw, 1944; Wu et al., 1992; Srivastava & Chandra, 2009; Deroin & Rakotondrainibe, 2015; Nopun et al., 2016; Becari-Viana & Schwartsburd, 2017). The adventitious roots of pteridophytes possess a primary xylem and phloem in the stele, a cortex with an endodermis and/or lacking an exodermis with Casparian bands, and lignified cortical walls (Chapple & Peterson, 1987; Damus et al., 1997; Schneider, 1997). The leaves have an endodermis, a sclerenchyma layer, and a cuticle as apoplastic barriers (Ribeiro, Santos & Moraes, 2007; De los Ángeles Lagoria et al., 2018; Mahley et al., 2018; Palacios-Rios et al., 2019). Moreover, the morphologies of sun and shade plants are obviously different (Boardman, 1977; Nasrulhaq-Boyce & Mohamed, 1987; Givnish, 1988; Santos-Silva, Mastroberti & De Araujo Mariath, 2011), the leaves of shade plants are large, with large cells, few mesophyll tissues, and decreased stomatal and vein densities (Ribeiro, Santos & Moraes, 2007; Zhang & Wu, 2013; Neira et al., 2017; Dematteis et al., 2019; Shah et al., 2019; Baer, Wheeler & Pittermann, 2020). In ferns, gymnosperms, and angiosperms, the endodermis or circumendodermal band and the exodermis act as barriers, which restrict water-solute exchange, reduce oxygen loss after submersion, and terrestrial environments (Ogura, 1972; Lersten, 1997; Enstone, Peterson & Ma, 2003; Hernandez-Hernandez et al., 2012; Geldner, 2013; Xiang et al., 2019; Yang et al., 2011, 2014, 2019a, 2019b, 2019c, 2020; Zhang et al., 2017; Zhang, Yang & Seago, 2018).

Phylogenetic and ontogenetic relationships within *Adiantum* have historically been based on anatomical characteristics, particularly the presence of dictyostele in the leaves (Wylie, 1949; Imaichi, 1988; Huiet et al., 2018). However, recent molecular genetic analyses have suggested that *A. reniforme* var. *sinense* is synonymous with *A. nelumboides*, in opposition to classical morphological taxonomy (Lin, 1980; Zhang et al., 2013; Wang et al., 2015). In addition, although *A. reniforme* var. *sinense* has been well studied with respect to optimal spore culture conditions, structural sporophyte anatomy and photosynthetic capacity have received little attention (Wu et al., 2010, 2011; Liao et al., 2007).

Here, we aimed to investigate the structural and histochemical features of the *A. reniforme* var. *sinense* sporophyte to determine whether these features were adaptations to dry cliff environments. We also sampled the leaves of *A. reniforme* var. *sinense* sporophytes in the sun and shade to identify the morphological and anatomical traits that indicated adaptations to shady environments; the leaves exhibited the same traits observed in other species. Evidence of such adaptive characteristics might help to explain the ability of *A. reniforme* var. *sinense* to grow in shady, dry cliff environments. Our results may also inform future studies of the ex situ conservation, taxonomy, evolution and phylogeny of this rare plant and its relatives.

## Materials and Methods

### Plant sourcing and collection

*Adiantum reniforme* var. *sinense* specimens were cultivated at the Chinese Germplasm Resource Nursery of the Three Gorges Corporation (Ichang, Hubei, China). Several sporophytes were collected in October 2019. From each plant, we collected approximately 10 roots, eight rhizomes, five leaves growing in the sun (intensity of illumination about 4,840 lux; humidity 51.6%) and five leaves growing in the shade (intensity of illumination about 805.5 lux; humidity 49.5%). Freshly collected samples were immediately fixed in formaldehyde-alcohol-acetic acid (FAA) (Ruzin, 1999).

### Microstructure and histochemistry

Root tissues were then sectioned freehand, using a two-sided razor blade, under a stereoscope (JNOEC JSZ6, China). Root sections were cut at 5, 10 and 20 mm from the root tip. Rhizomes were cut into two sections: young (~10 mm from the tip; white surface coloration) and mature (~30 mm from the tip; brown surface). Petioles were also cut into young (white surface) and mature (black surface) sections. Blades were cut in the center to determine tissue thickness. Sections were divided into three sets such that each set contained 3–6 sections (obtained from different specimens) representing each distance from the root tip; the young and mature rhizomes; the young and mature petioles; and the blade centers and margins.

Each set of sections was stained with one of three stains: 0.1% (w/v) Sudan red 7B (SR7B) to test for suberin in the cell walls (Brundrett, Kendrick & Peterson, 1991); 0.1% (w/v) berberine hemisulfate-aniline blue (BAB) to test for Casparian bands and lignin in the cell walls (Brundrett, Enstone & Peterson, 1988; Seago et al., 1999); and 0.05% (w/v) toluidine blue O (TBO) to visualize tissue structures (Feder & O’Brien, 1968; Peterson, Peterson & Melville, 2008). All specimens were washed with sterile water 2–3 times, mounted with sterile water, and then examined using bright-field microscopy under a Leica DME microscope (Germany). Specimens were photographed with a digital camera and a micrometer (Nikon E5400, Japan). Specimens stained with BAB were viewed under ultraviolet light on an Olympus IX71 epifluorescence microscope with excitation filter G 365 nm, absorption filter barriers U-WB (blue light), dichromatic mirror DM 500, compensation excitation filter BP 450–480, and compensation absorption filter BA 515, BAB-stained specimens were photographed with a digital camera and a micrometer (RZ200C-21; Ruizhi Cop., China) (Yang et al., 2011).

### Blade data collection and statistical analyses

The length and width of leaf area was measured with a centimeter ruler. Tissue thickness data was collected from sections stained by SR7B, BAB and TBO as discussed in the above section. We sectioned the leaflet at the blade margin to measure the density of the fine veins. We also sectioned the leaflet at the blade center (not along the blade margin) to measure the stoma and cell number and size of tissue density and epidermal features. All sections included five blade samples that remained unstained and were mounted with sterile water. Specimens were observed under a Leica DME microscope with a micrometer. Differences between the morphological and anatomical traits from sunny and shady blades were analyzed with the paired-samples *T*-test using SPSS (version 13.0; SPSS Inc., Chicago, IL, USA).

## Results

**Figure 1:**
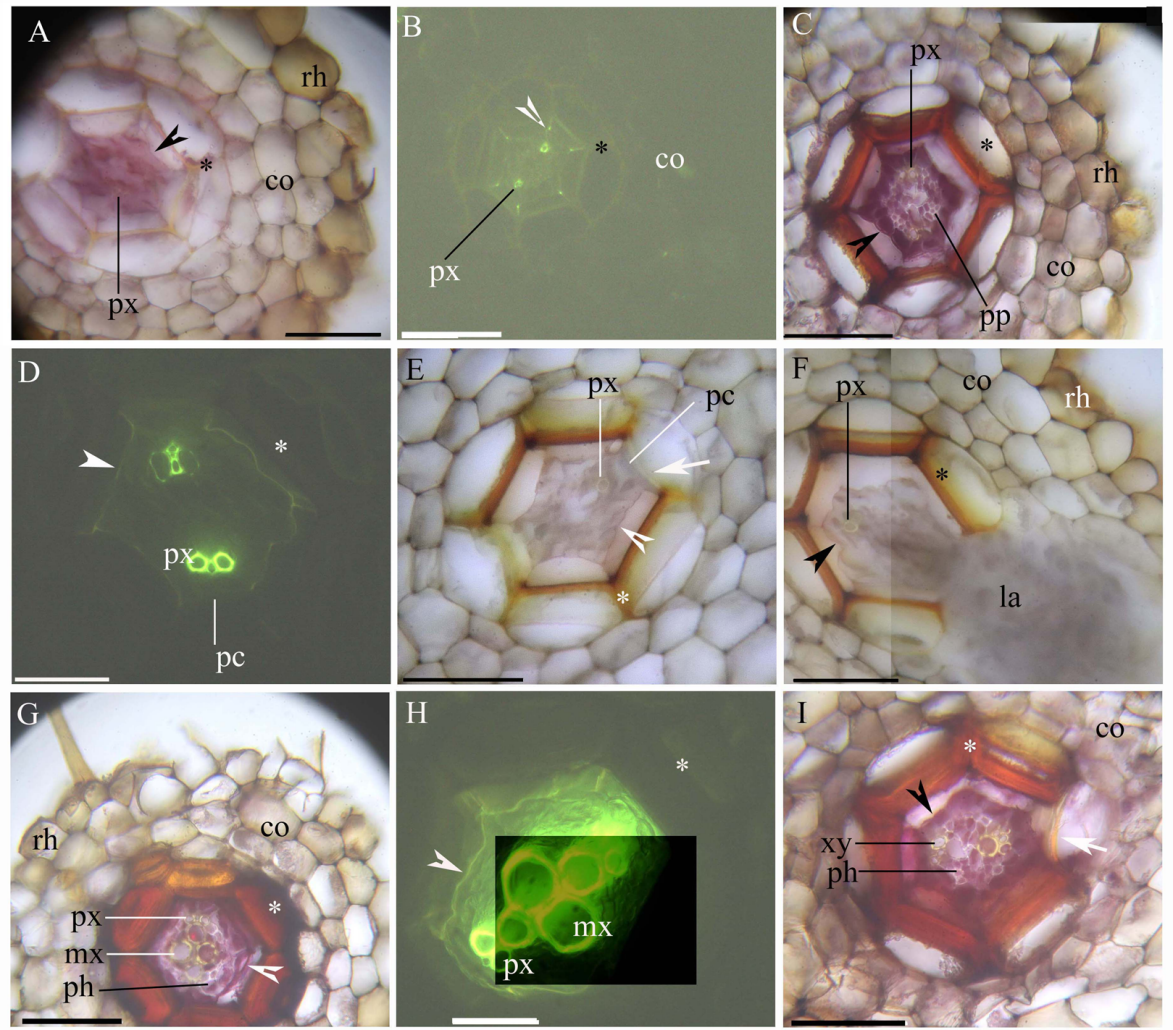
(A–I) A Photomicrographs of the adventitious roots of *Adiantum reniforme* var. *sinense* (70–130 mm long); scale bars = 50 μm. (A) A total of 5 mm from root tip. Protoxylem, endodermis (arrowhead), sclerenchyma layer (*), cortex, rhizodermis. Staining: Berberine sulfate-aniline blue (BAB); (B) A total of 5 mm from root tip. Protoxylem, endodermis (arrowhead), sclerenchyma layer (*), cortex. Staining: BAB; (C) A total of 10 mm from root tip. Protoxylem, protophloem, suberized endodermis (arrowhead), sclerenchyma layer (*), cortex, rhizodermis. Staining: Sudan red 7B (SR7B); (D) A total of 10 mm from root tip. Protoxylem, suberized endodermis (arrowhead), passage cells, sclerenchyma layer (*). Staining: BAB; (E) A total of 10 mm from root tip. Protoxylem, endodermis (arrowhead), passage cells, sclerenchyma layer (*), idioblast (arrow), cortex. Staining: SR7B; (F) A total of 10 mm from root tip. Protoxylem, suberized endodermis (arrowhead), sclerenchyma layer (*), cortex, lateral root, rhizodermis. Staining: SR7B; (G) A total of 20 mm from root tip. Protoxylem, metaxylem, primary phloem, suberized endodermis (arrowhead), sclerenchyma layer (*), cortex, rhizodermis. Staining: SR7B; (H) A total of 20 mm from root tip. Protoxylem, metaxylem, suberized endodermis (arrowhead), sclerenchyma layer (*). Staining: BAB; (I) A total of 20 mm from root tip. Primary xylem, primary phloem, suberized endodermis (arrowhead), sclerenchyma layer (*), idioblast (arrow), cortex. Staining: SR7B; Abbreviations: co, cortex; la, lateral root; mx, metaxylem; pc, passage cells; ph, primary phloem; pp, protophloem; px, protoxylem; rh, rhizodermis; xy, primary xylem.

The stele within the adventitious roots had diarch symmetry with protoxylem poles (Figs. 1A–1I). At 5 mm from the root tip, we observed faint Casparian bands in the endodermis of the inner root cortex, a thin-walled sclerenchyma layer around the endodermis, and a rhizodermis on the root surface (Figs. 1A and 1B). At 10 mm from the root tip, lateral roots emerged from the stele, and the stele had prominent protoxylem and protophloem (Figs. 1C–1F). In addition, the endodermis had complete suberin lamellae with a few passage cells, and the sclerenchyma layer had thicker walls (except for the idioblasts) opposite the passage cells and the protoxylem (Figs. 1C–1F). At 20 mm from the root base, the stele had primary xylem and phloem tissues, as well as deep suberin lamellae in the endodermis; the sclerenchyma layer was thick-walled (Figs. 1G–1I).

**Figure 2:**
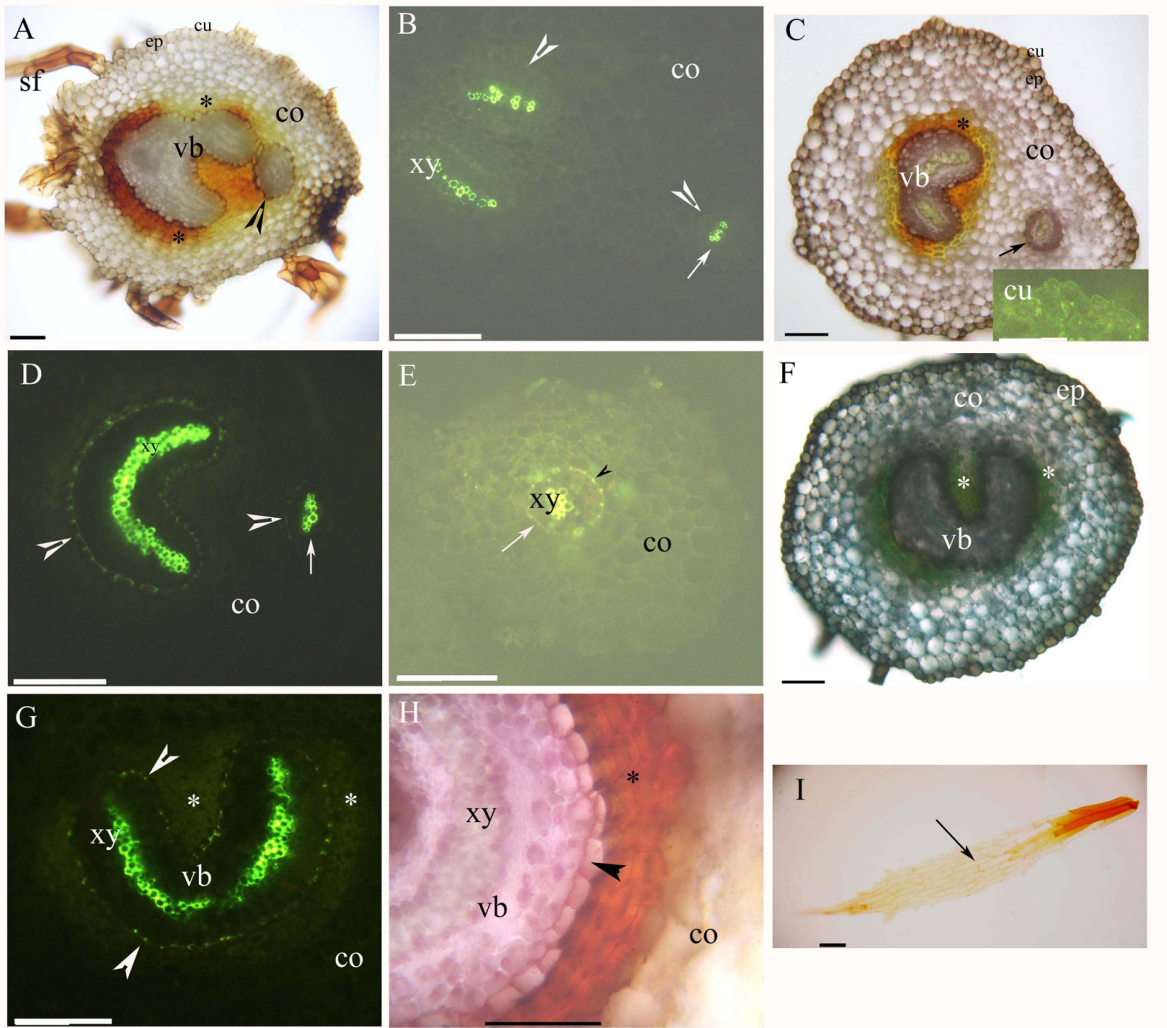
(A–I) Photomicrographs of the young (A and B) and mature (C–I) rhizomes of *A. reniforme* var. *sinense* (40–60 mm long); scale bars = 50 μm. (A) Vascular bundles, immature sclerenchyma layer (*), petiole vascular bundles (arrowhead), cortex, epidermis, cuticle, scale leaves. Staining: Sudan red 7B (SR7B); (B) Primary xylem, endodermis (arrowhead), petiole vascular bundles (arrow), cortex. Staining: Berberine sulfate-aniline blue (BAB); (C) Vascular bundles, sclerenchyma layer (*), petiole vascular bundles (arrow), cortex, epidermis, cuticle. Staining: SR7B. Inset: young cuticle. Staining: BAB; (D) Primary xylem, suberized endodermis (arrowhead), petiole vascular bundles (arrow), cortex. Staining: BAB; (E) Petiole vascular bundles (arrow), primary xylem, suberized endodermis (arrowhead), cortex. Staining: BAB; (F) Vascular bundles, sclerenchyma layer (*), cortex, epidermis. Staining: TBO; (G) Vascular bundles, primary xylem, suberized endodermis (arrowhead), sclerenchyma layer (*), cortex. Staining: BAB; (H) Vascular bundles, primary xylem, suberized endodermis (arrowhead), sclerenchyma layer (*), cortex. Staining: SR7B; (I) Scale leaves (arrow). Unstained. Abbreviations: co, cortex; cu, cuticle; ep, epidermis; sf, scale leaf; vb, vascular bundles; xy, primary xylem.

Both young and mature rhizomes had a dictyostele surrounded by sclerenchyma layers. The dictyostele included petiole vascular bundles with a prominent central protoxylem (Figs. 2A–1H). Each vascular bundle had an endodermis with Casparian bands (Figs. 2B, 2D, 2E and 2G), which became suberized at maturity (Figs. 2D, 2E, 2G and 2H). The rhizomes had a parenchymatous cortex; the rhizome surface had a thin cuticle and brown scale leaves (Figs. 2A, 2C, 2F and 2I). The petiole vascular bundles originated from the rhizomes (Figs. 2A–2E).

**Figure 3:**
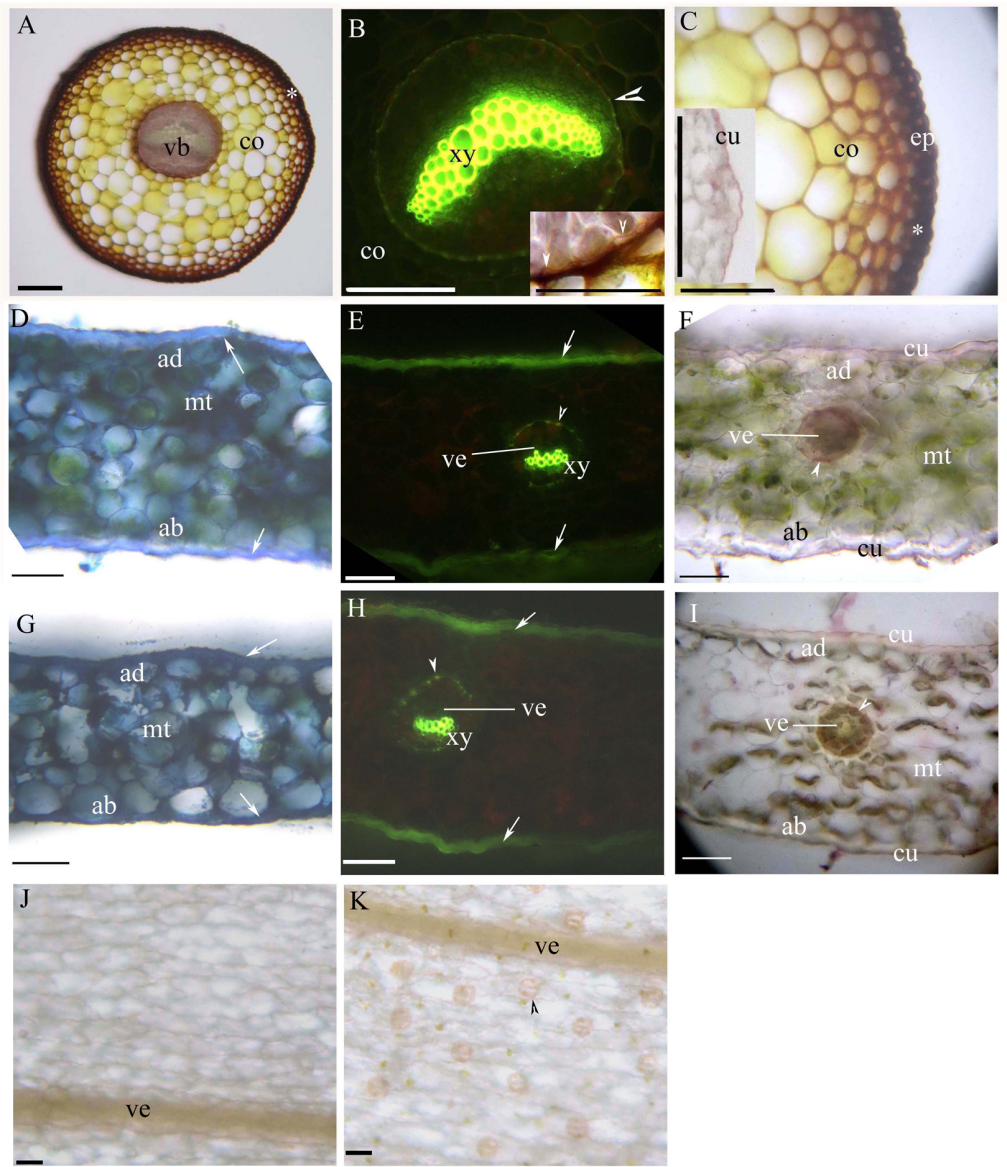
(A–K) Photomicrographs of the mature petioles (A–C), sunny leaves (D–F) and shady leaves (G–K) of *A. reniforme* var. *sinense*; scale bars = 50 μm. (D–I) Show the leaf blade adaxial side up. (A) Vascular bundles (arrowhead), cortex, peripheral sclerenchyma layer (*). Staining: Sudan red 7B (SR7B); (B) Primary xylem, suberized endodermis (arrowhead), cortex. Staining: Berberine sulfate-aniline blue (BAB). Inset shows the suberized endodermis (arrowhead). Staining: SR7B; (C) Cortex, peripheral sclerenchyma layer (*), epidermis. Inset shows the cuticle of a young petiole. Staining: SR7B; (D) Isolateral mesophyll tissue, hypodermal sterome (arrow), adaxial epidermis, abaxial epidermis. TBO; (E) Vein, primary xylem, suberized endodermis (arrowhead), hypodermal sterome (arrow). Staining: BAB; (F) Vein, isolateral mesophyll tissue, suberized endodermis (arrowhead), adaxial epidermis, abaxial epidermis, cuticle. Staining: SR7B; (G) Isolateral mesophyll tissue, hypodermal sterome (arrow), adaxial epidermis, abaxial epidermis. TBO; (H) Vein, primary xylem, suberized endodermis (arrowhead), hypodermal sterome (arrow). Staining: BAB; (I) Vein, isolateral mesophyll tissue, suberized endodermis (arrowhead), adaxial epidermis, abaxial epidermis, cuticle. Staining: SR7B; (J) Adaxial surface lack stomata, vein. Unstaining; (K) Abaxial surface contain stomata (arrowhead), vein. Unstaining; Abbreviations: ad, adaxial epidermis; ab, abaxial epidermis; co, cortex; cu, cuticle; ep, epidermis; mt, isolateral mesophyll tissue; vb, vascular bundles; ve, vein; xy, primary xylem.

Young and mature petioles had a single vascular bundle with a central endodermis, a cortex, a peripheral sclerenchyma layer and an epidermis (Figs. 3A–3C). At maturity, the endodermis had Casparian bands and suberized lamellae (Fig. 3B). The petiole surface had a thin cuticle (Fig. 3C). Table 1 shows the morpho-anatomical characteristics of the blades: leaf area, tissue thicknesses, tissue densities, and epidermal features. The leaf blades from both sunny and shady environments had an epidermis, isolateral mesophyll tissue, and a dichotomous vein with a sclerenchyma layer (Figs. 3D–3I; Table 1); the endodermis of the dichotomous vein had Casparian bands and suberized lamellae (Figs. 3E, 3F, 3H and 3I). The stoma was only present on the abaxial epidermis (Figs. 3J and 3K); the shady blades had fewer and larger stoma compared to the sunny blades (Table 1). The thin cuticle was slightly thicker on the adaxial and abaxial on the sunny blades compared to shady blades (Figs. 3D–3I; Table 1). The surface of the leaf blade had a hypodermal sterome (Figs. 3D–3I). Lastly, the mesophyll was thicker in the middle of the sunny blades than the shady blades (Figs. 3D–3I; Table 1).

**Table 1:** Blades morphological and anatomical traits and environmental data.

Morphological traits	Sunny (mean ± SE)	Shady (mean ± SE)
Leaf area		
Length (cm)	4.64 ± 0.31	4.70 ± 0.28
Width (cm)	3.88 ± 0.36	4.16 ± 0.36
Thickness (μm)	208.96 ± 23.77	189.60 ± 10.06
Tissue thickness		
Adaxial cuticle (μm)	1.5 ± 0.41	1.22 ± 0.23
Abaxial cuticle (μm)	0.54 ± 0.11	0.30 ± 0.10*
Mesophyll (μm)	153.60 ± 15.18	129.40 ± 11.17*
Adaxial epidermis (μm)	13.06 ± 4.16	10.64 ± 2.60
Abaxial epidermis (μm)	15.36 ± 4.90	13.42 ± 1.39
Adaxial hypodermal sterome (μm)	11.14 ± 2.50	10.62 ± 2.75
Abaxial hypodermal sterome (μm)	5.38 ± 2.50	6.24 ± 0.83
Tissue density		
Adaxial epidermis (n mm^2^)	282.40 ± 25.83	281.20 ± 22.79
Abaxial epidermis (n mm^2^)	188.60 ± 27.82	171.40 ± 10.29
Abaxial stomatal (n mm^2^)	45.80 ± 4.82	29.80 ± 3.11*
Marginal of fine veins (n mm)	1.90 ± 0.20	2.04 ± 0.33
Epidermal features		
Adaxial epidermis length (μm)	88.00 ± 23.32	88.80 ± 2.28
Adaxial epidermis width (μm)	40.00 ± 5.66	44.80 ± 7.29
Abaxial epidermis length (μm)	129.60 ± 39.35	105.20 ± 15.85
Abaxial epidermis width (μm)	43.20 ± 7.16	47.00 ± 4.69
Abaxial stomatal length (μm)	36.80 ± 4.38	38.40 ± 3.58
Abaxial stomatal width (μm)	30.40 ± 3.58	36.80 ± 4.38*

**Note:**

* A significant difference according to *T*-test (*P* < 0.05).

## Discussion

We observed various morphological and anatomical characteristics that were likely to support the successful colonization of dry and shady environments by *A. reniforme* var. *sinense*. For example, in *A. reniforme* var. *sinense*, the adventitious roots, the rhizomes, and the leaf axes all had an endodermis with the following histochemical features; Casparian bands and suberin lamellae surrounded by sclerenchyma layers. These characteristics tend to vary distinctly across tissues and organs in almost all other vascular plants (Ogura, 1972; Fahn, 1990; Lersten, 1997; Enstone, Peterson & Ma, 2003; Evert, 2006; Yang et al., 2011, 2014, 2019a, 2019b, 2019c, 2020; Zhang et al., 2017; Zhang, Yang & Seago, 2018; Crang, Lyons-Sobaski & Wise, 2018; Xiang et al., 2019). Displaying alike to *A. reniforme* var. *sinense*, the terrestrial species *Asplenium* sp. and the epiphytic *Pleopeltis macrocarpa* possess an endodermis and a sclerenchyma layer around the vascular bundles throughout the plant (Wetzel et al., 2017; De los Ángeles Lagoria et al., 2018). The endodermis and the sclerenchyma layers support and protect the bodies of vascular plants such as ferns, gymnosperms, and angiosperms (Fahn, 1990; Lersten, 1997; Enstone, Peterson & Ma, 2003; Evert, 2006; Yang et al., 2011, 2014, 2019a, 2019b, 2019c, 2020; Crang, Lyons-Sobaski & Wise, 2018; Zhang et al., 2017; Zhang, Yang & Seago, 2018; Xiang et al., 2019). The presence of these structures suggested that *A. reniforme* var. *sinense* would survive in dry cliff environments (Chapple & Peterson, 1987; Lersten, 1997; Yang et al., 2011, 2014, 2019a, 2019b, 2019c, 2020; Neira et al., 2017; Wetzel et al., 2017; Zhang et al., 2017; Zhang, Yang & Seago, 2018; De los Ángeles Lagoria et al., 2018; Xiang et al., 2019).

Many pteridophytes have an endodermis and a sclerenchyma layer in the adventitious roots, including the terrestrial species *Platycerium bifurcatum* (Chapple & Peterson, 1987), *Asplenium* sp. (Schneider, 1997; Leroux et al., 2011; Wetzel et al., 2017), *Lycopodium obscururn, Selaginella* sp. (Damus et al., 1997), *Pteris vittata* (Bondada, Tu & Ma, 2006; Sridhar et al., 2011), *Pleopeltis* sp. (Hernandez et al., 2013; De los Ángeles Lagoria et al., 2018) and *Doryopteris triphylla* (Neira et al., 2017). The roots of these plants had several additional similarities to those of *A. reniforme* var. *sinense*. For example, the endodermis of *Platycerium bifurcatum* deposited suberin lamellae (Chapple & Peterson, 1987); idioblasts were identified in the sclerenchyma layer in the *Platycerium bifurcatum* and *Pleopeltis macrocarpa* (Chapple & Peterson, 1987; De los Ángeles Lagoria et al., 2018); the roots of *Selaginella* sp. had an exodermis and lignified cortical walls (Damus et al., 1997); the roots of *Platycerium bifurcatum*, *Pleopeltis* sp., and *Doryopteris triphylla* had two or more sclerenchyma layers (Chapple & Peterson, 1987; Hernandez et al., 2013; Neira et al., 2017; De los Ángeles Lagoria et al., 2018); and the rhizodermis of *Asplenium* sp. had helical thickenings (Wetzel et al., 2017). Compared to these species, *A. reniforme* var. *sinense* roots weakly adapted to dry environments (Chapple & Peterson, 1987; Damus et al., 1997; Hernandez et al., 2013; Neira et al., 2017; Wetzel et al., 2017; De los Ángeles Lagoria et al., 2018).

In *A. reniforme* var. *sinense*, the rhizomes had a dictyostele and an endodermis surrounded by sclerenchyma layers and a thin cuticle. The rhizome structures presented similarities to terrestrial ferns such as *Onoclea sensibilis* (Wardlaw, 1944), *Polypodium* sp. (Srivastava & Chandra, 2009), *Pteris vittata* (Sridhar et al., 2011), *Ceradenia* sp. (Deroin & Rakotondrainibe, 2015), *Blotiella lindeniana* (Becari-Viana & Schwartsburd, 2017) and *Doryopteris triphylla* (Neira et al., 2017). In contrast, epiphytic and xerophytic species, such as *Asplenium* sp., *Pleopeltis macrocarpa* and *Niphobolus adnascens*, had peripheral sclerenchyma layers under the epidermis. Thus, these plants were more adapted to xerophytic environments than shady *A. reniforme* var. *sinense* (Pande, 1935; Wetzel et al., 2017; De los Ángeles Lagoria et al., 2018).

The petioles of *A. reniforme* var. *sinense* had a central endodermis and a peripheral sclerenchyma layer underneath the epidermis, which is similar to other ferns (Hernandez-Hernandez et al., 2012), including *Anemia* (Ribeiro, Santos & Moraes, 2007), *Pteris* (Bondada, Tu & Ma, 2006; Martínez & Vilte, 2012; Sridhar et al., 2011; Palacios-Rios et al., 2019), *Davallia* (Ummu-Hani et al., 2013), *Blechnum* (Noraini et al., 2014), *Asplenium* (Wetzel et al., 2017), *Doryopteris triphylla* (Neira et al., 2017) and *Pleopeltis macrocarpa* (De los Ángeles Lagoria et al., 2018). In *Asplenium* species, the petiole endodermis was surrounded a sclerenchyma layer (Wetzel et al., 2017), whereas in *Doryopteris triphylla*, the petiole epidermis had a thick cuticle at the surface (Neira et al., 2017).

The blades of *A. reniforme* var. *sinense* had suberized endodermises, with Casparian strips around the vein and a hypodermal sterome (Mahley et al., 2018). These structures were like those of *Anemia* sp. (Ribeiro, Santos & Moraes, 2007; Mahley et al., 2018), indicating that *A. reniforme* var. *sinense* adapted to terrestrial environments. The blades of *A. reniforme* var. *sinense* had isolateral mesophyll, which was also identified in *Coptis chinensis*, *Doryopteris pentagona*, *Lygodium japonicum, Pteris multifida*, *Nephrolepis cordifolia*, *A. capillus-veneris* and *Pteris ensiformis* cv. *victoriae* (Yuan, Zhang & Shang, 2007; Zhang & Wu, 2013; Dematteis et al., 2019). Blades with isolateral mesophyll are a shared feature among the sciophytes (Yuan, Zhang & Shang, 2007; Zhang & Wu, 2013; Dematteis et al., 2019). The blades of *A. reniforme* var. *sinense* had thin cuticles, which is like to other shade ferns such as *Lygodium japonicum, Pteris multifida*, *Nephrolepis cordifolia*, *A. capillus-veneris, Pteris ensiformis* cv. *victoriae* and *Doryopteris pentagona* (Zhang & Wu, 2013; Dematteis et al., 2019). The thin cuticles of the *A. reniforme* var. *sinense* blades suggested that this species was adapted to shady environments, in contrast to sunny or xeromorphic ferns, such as *Cheilanthes glauca* and *Doryopteris triphylla*, which have thick cuticles (Zhang & Wu, 2013; Neira et al., 2017; Dematteis et al., 2019; Shah et al., 2019). In *A. reniforme* var. *sinense*, the shaded blades had fewer mesophyll tissues and lower stomatal densities than the unshaded blades, showing that this species displays environmental adaptive plasticity (Givnish, 1988; Ribeiro, Santos & Moraes, 2007; Santos-Silva, Mastroberti & De Araujo Mariath, 2011; Neira et al., 2017; Dematteis et al., 2019; Shah et al., 2019; Baer, Wheeler & Pittermann, 2020).

## Conclusion

The adventitious roots, petioles, and rhizomes of *A. reniforme* var. *sinense* had several structures that adapted to dry environments, including an endodermis, sclerenchyma layers, and hypodermal sterome (Chapple & Peterson, 1987; Neira et al., 2017; Wetzel et al., 2017; De los Ángeles Lagoria et al., 2018). However, like many shade-adapted ferns (Evert, 2006; Yuan, Zhang & Shang, 2007; Zhang & Wu, 2013; Crang, Lyons-Sobaski & Wise, 2018), this plant also had a thin cuticle and isolateral mesophyll, which suggested a tolerance of shady environments. In contrast, epiphytic and xerophytic ferns, which are more adapted to xeric environments but also do not tolerate shade, have roots with an exodermis, many sclerenchyma layers, and helical thickenings (Chapple & Peterson, 1987; Damus et al., 1997; Neira et al., 2017; Wetzel et al., 2017; De los Ángeles Lagoria et al., 2018); rhizomes with peripheral sclerenchyma layers (Wetzel et al., 2017; De los Ángeles Lagoria et al., 2018); and leaves with thick cuticles (Neira et al., 2017). Thus, the anatomical structures of the sciophyte *A. reniforme* var. *sinense* identified herein were consistent with adaptations to dry and shady environments.

## Supplemental Information

10.7717/peerj.9937/supp-1 Supplemental Information 1 Raw Data: Leaf morphological and anatomical traits and environmental data.

## Additional Information and Declarations

### Data Availability

The following information was supplied regarding data availability: The raw data is in Figs. 1–3 and a Supplemental File.
